# Celecoxib Ameliorates Non-Alcoholic Steatohepatitis in Type 2 Diabetic Rats via Suppression of the Non-Canonical Wnt Signaling Pathway Expression

**DOI:** 10.1371/journal.pone.0083819

**Published:** 2014-01-03

**Authors:** Feng Tian, Ya Jie Zhang, Yu Li, Ying Xie

**Affiliations:** 1 Department of Gastroenterology, Shengjing Hospital of China Medical University, Shenyang, Liaoning Province, China; 2 Department of Gastroenterology, Third People's Hospital of Dalian city, Dalian, Liaoning Province, China; The University of Hong Kong, Hong Kong

## Abstract

Our aim was to test whether pharmacological inhibition of cycloxygenase-2 (COX-2) reverses non-alcoholic steatohepatitis (NASH) in type 2 diabetes mellitus (T2DM) rats via suppression of the non-canonical Wnt signaling pathway expression. Twenty-four male Sprague-Dawley rats were randomly distributed to two groups and were fed with a high fat and sucrose (HF-HS) diet or a normal chow diet, respectively. After four weeks, rats fed with a HF-HS diet were made diabetic with low-dose streptozotocin. At the 9^th^ week the diabetic rats fed with a HF-HS diet or the non-diabetic rats fed with a normal chow diet were further divided into two subgroups treated with vehicle or celecoxib (a selective COX-2 inhibitor, 10 mg/Kg/day, gavage) for the last 4 weeks, respectively. At the end of the 12^th^ week, rats were anesthetized. NASH was assessed by histology. Related cytokine expression was measured at both the protein and gene levels through immunohistochemistry (IHC), Western blot and real-time PCR. T2DM rats fed with a HF-HS diet developed steatohepatitis and insulin resistance associated with elevated serum alanine aminotransferase (ALT), aspartate aminotransferase (AST), insulin levels and the non-alcoholic fatty liver disease (NAFLD) activity score (NAS). The expression of Wnt5a, JNK1, NF-κB p65, and COX-2 were all significantly increased in the T2DM-NASH group compared with the control and control-cele group. Hepatic injury was improved by celecoxib in T2DM-NASH-Cele group indicated by reduced serum ALT and AST levels and hepatic inflammation was reduced by celecoxib showed by histology and the NAFLD activity score (NAS). Serum related metabolic parameters, HOMA-IR and insulin sensitivity index were all improved by celecoxib. The expression of Wnt5a, JNK1, NF-κB p65, and COX-2 expression were all suppressed by celecoxib in T2DM-NASH-Cele group. The results of the present study indicated that celecoxib ameliorated NASH in T2DM rats via suppression of the non-canonical Wnt5a/JNK1 signaling pathway expression.

## Introduction

Non-alcoholic steatohepatitis (NASH) is one of the most frequent causes of abnormal liver function and correlates with central adiposity, insulin resistance, dyslipidemia, and T2DM [1]. In diabetes patients, NASH is more frequent than in the general people by about 3-fold [2]. T2DM might be an independent risk factor for the progression of non-alcoholic fatty liver disease (NAFLD) from simple steatosis to NASH and fibrosis [3]–[4]. The mechanisms by which diabetes aggravates the progression of NASH mainly include insulin resistance, insulin deficiency, inflammatory response, and oxidative stress mediated by cytokines and chemokines [4]–[5]. However, the role of cytokines and chemokines on the development of NASH remains poorly understood and therapeutic options are limited.

Growing evidence support the notion that among the pro-inflammatory mediators that have been upregulated in NAFLD is cyclooxygenase2 (COX-2). Accordingly, NASH development can be protected against by treatment with COX-2 inhibitors [6]–[9]. Hepatic nuclear factor-κB (NF-κB) with downstream consequences including COX-2, TNF-α, and IL-6 were increased and aggravated inflammation in NASH [6]–[7]. COX-2 inhibitors reduced COX-2 expression by inhibiting NF-κB expression and activation [10]. Despite of these studies, there was hardly any research which indicated the role of COX-2 in the diabetic model of NASH and whether inhibiting COX-2 reversed diabetes-related NASH.

Recent evidence has implicated a direct role of disturbed Wnt signaling on metabolic syndrome including obesity, insulin resistence and T2DM [11]. The Wnt signaling pathways are involved the process of metabolic disorders including inflammatory response and insulin resistance [11]. However, studies about the role of Wnt pathway in hepatic metabolism has been largely ignored, especially for the non-canonical Wnt pathway. Hepatocyte-specific loss of β-catenin led to increased susceptibility to developing steatohepatitis and fibrosis in the mice fed with the MCD diet [12], which suggested the protective effect of the canonical Wnt/β-catenin signaling. Although Ouchi et al [13] showed that increased non-canonical Wnt5a/JNK1 pathway expression in adipose tissue led to enhanced insulin resistance and fatty liver, the influence of hepatic Wnt5a signaling pathway on NASH is still not researched and remains unknown at present.

As shown by recent reports, Wnt5a can induce NF-κB activity and NF-κB can upregulate Wnt5a expression [14]–[15]. COX-2 can be upregulated by NF-κB [7] and COX-2 inhibitors can inactivate NF-κB pathway [10]. These studies prompt the interaction among these cytokines. All of findings above triggered us to clarify the pathogenetic role exerted by the non-canonical Wnt5a/JNK1 pathway, NF-κB, and COX-2 in NASH development. We investigated their protein and mRNA levels in liver tissues. And we preliminarily analysed whether there was interaction among these cytokines. Ultimately we aimed to determine if the protective effect of the selective COX-2 inhibitor, celecoxib, on T2DM-related NASH was associated with expression changes of these cytokines.

## Methods

### Ethics Statement

All animal experimental protocols were approved by the ethics committee of Shengjing Hospital of China Medical University and conducted in accordance with their guidelines.

### Animals and experimental design

Twenty-four male Sprague Dawley rats were purchased from China Medical University at 5–6 weeks of age. After one week acclimatization, rats were randomly distributed into two groups: (1) control rats were fed with a normal chow diet (CHOW; 5%kcal fat, with total calorific value 25 kJ/kg) (n = 12), and (2) rats were fed with a high fat and sucrose diet (HF-HS; 33%kcal fat, 54%kcal carbohydrate, with total calorific value 41.4 kJ/kg) (n = 12). After four weeks of diets, the HF-HS group were rendered T2DM with low dose streptozotocin (STZ; Sigma) intraperitoneally (30 mg/kg of body weight). After one week, blood glucose was measured from tail vein using point of care blood glucose monitoring system(Accu-Chek Advantage, Roch Diagnostics, USA). The rats with blood glucose <16.7 mmol/L were injected with STZ again (30 mg/kg). The blood glucose and body weight were measured every week. Four weeks following the STZ injections, rats with blood glucose levels of ≥16.7 mmol/L were considered diabetic. In order to determine the capacity of celecoxib to prevent NASH, four subgroups were formed and treated respectively with vehicle (saline, gavage, daily) as the control group or the T2DM-NASH group and celecoxib (10 mg/kg/, gavage, daily; Pfizer, New York, NY) as the control-Cele group or the T2DM-NASH-Cele group for four weeks. At the end of the 12^th^ week, rats were anesthetised with chloral hydrate (3 ml/kg of body weight) and terminated by exsanguination.

### Blood sampling and analysis

Blood was obtained by abdominal aortic puncture and separated by centrifugation (1500 rpm, 20 min) for serum. Serum alanine aminotransferase (ALT), aspartate aminotransferase (AST), glucose, low-density lipoprotein (LDL), and high-density lipoprotein (HDL) concentrations were determined using an automatic biochemical analyzer (ci16200, Abbott, USA). Serum insulin was determined with an Insulin Radioimmunoassay Kit (HTA Co. Ltd, People's Republic of China).

### Assessment of insulin resistance

To assess insulin sensitivity, insulin tolerance test (ITT) was conducted at 11 weeks. Rats fasted for 6 h were injected i.p. with rh insulin (0.5 U/kg of body weight). Blood glucose was measured from tail vein using point of care blood glucose monitoring system (Accu-Chek Advantage, Roch Diagnostics, USA) at 0, 30, 60, 90, and 120 min after insulin administration. The change in Area Under the Curve (AUC) was compared with the AUC obtained for the T2DM-NASH group (normalized to 100%). The homeostasis model assessment of insulin resistance (HOMA-IR) method was used to calculate insulin resistance as [fasting insulin (mIU/L)×fasting glucose (mmol/L)]/22.5. The insulin sensitivity index (ISI) was assessed by ln {1/[fasting insulin (mIU/L)×fasting glucose (mmol/L)]}.

### Histological examination

Livers were fixed in 10% buffered formalin, processed, and embedded in paraffin for Hematoxylin–Eosin staining (H&E). Histological scoring was graded by pathologists (blinded to the goups) for hepatic steatosis, lobular inflammation, ballooning, and fibrosis in accordance with the 2011 programme for diagnosis and treatment of non-alcoholic steatohepatitis called the NAFLD activity score (NAS), issued by the USA CRN [16]. NASH was diagnosed if NAS (total scores) was greater than four.

### Immunohistochemistry(IHC)

Immunostaining was performed on 3 µm sections after deparaffinization. Microwave or high pressure antigen retrieval was performed in citrate buffer pH 6.0 for 10 min prior to peroxidase quenching with 3% H_2_O_2_ in phosphate-buffered saline (PBS) for 10 min. The sections were then washed in water and preblocked with normal goat or rabbit serum for 10 min. Then, slides were incubated, respectively, with anti-Wnt5a (Abcam, USA), anti-JNK1 (Santa Cruz Biotechnology, USA), anti-NF-κB p65 (Santa Cruz Biotechnology, USA), and anti-COX-2 (Santa Cruz Biotechnology, USA) (final concentrations 1∶100, 1∶100, 1∶200, 1∶320, respectively) for over-night at 4°C. The sections were then incubated with biotinylated secondary antibodies (1∶400) for 20 min. Following a washing step with PBS, the avidin–biotin complex was applied. Finally, the sections were rinsed in PBS, developed with diaminoben-zidine tetrahydrochloride substrate for 3 min and counterstained with hematoxylin. Five fields with a final magnification of ×400 were randomly selected for each rat liver, and the Integral Optical Density(IOD) were analyzed by Image Pro Plus 6.0 software.

### Western blotting

Liver tissue was homogenized in RIPA Lysis Buffer containing PMSF (a protease inhibitor (Beyotime Institute of Biotechnology), and total protein concentrations were determined by bicinchoninic acid protein assay (Boster). About 50 micrograms of protein were loaded on 5–10% gradient gels. Proteins were transferred to a PVDF membrane(Thermo Scientific) for 70 min at 100 V. The membranes were incubated in blocking buffer for 2 hours before the addition of primary antibody including Wnt5a (Abcam, USA), JNK1 (Santa Cruz Biotechnology, USA), NF-κB p65 (Abcam, USA) and COX-2 (Proteintech Group, PTG, USA). All of the primary antibodies were used at a 1∶400 dilution. All secondary antibodies were diluted 1∶1000 except for anti-NF-κB p65, which was diluted 1∶3000. Proteins were detected via enhanced chemiluminescence (Beyotime Institute of Biotechnology), and the intensity of protein bands were quantified using the Quantity One software (Bio-Rad, USA).

### Quantitative real-time polymerase chain reaction

Total RNA was extracted from liver tissue (100 mg) with TRIzol reagent (Takara, Japan). RNA amount and quality was determined using a Nanodrop spectrophotometer. Total RNA (500 ng) was then reverse-transcribed to cDNA using the Reverse Transcription System from TaKaRa (Japan). cDNA (200 ng) was used in each PCR reaction. Real-time quantitative PCR was performed with an ABI Prism 7500 Sequence Detection System (Applied Biosystems, Foster City, CA) with SYBR Green reagents. The housekeeping gene β-actin was used as a reference gene for normalization. Primers sequences were as follows: Wnt5a: F: 5′-GCTTCAACTCCCCAACCA-3′, R: 5′-CTCG-CAGCCGTCCATC-3′; NF-κB p65: F: 5′-CGACGTATTGCTGTGCCTTC-3′, R: 5′-TTGAGAT-CTGCCCAGGTGGTA-3′; COX-2: F: 5′-CGGAGAGGAGAAGTGGGGTTTAG-3′, R: 5′-TGA-AAGAGGCGAAGGGACA-3′; β-actin: F: 5′-GGAGATTACTGCCCTGGCTCCTA-3′, R: 5′-GACTCATCGTACTCCTGCTTGCTG-3′. For each gene, relative change in steady state mRNA in samples was determined using the 2^−ΔΔCT^ method, corrected for the housekeeper.

### Statistical analysis

The results are presented as the mean±SD. Comparisons between two groups were analyzed via Student's *t*-test, and comparisons between more than two groups were analyzed via one-way ANOVA to identify differences among means. A probability of *P*<0.05 was taken to indicate a significant difference between means.

## Results

### Celecoxib reduced liver weight index and increased EAW/BW

Rats fed with a HF-HS diet showed significantly lower body weight (BW); but higher liver weight index than rats fed with a CHOW diet (Table 1). Although no significant difference between the T2DM-NASH group and T2DM-NASH-Cele group was found in BW and liver weights, the liver weight index did make a difference (Table 1). We observed increased central lipolysis in the T2DM-NASH group as shown by significantly lower EAW/BW, compared with the rats fed with a CHOW diet. In the T2DM-NASH-Cele group, celecoxib increased EAW/BW compared with the T2DM-NASH group (Table 1).

**Table 1:** Basic parameters in four groups.

	CHOW diet		HF-HS diet	
Group	control	control-cele	T2DM-NASH	T2DM-NASH-Cele
Body weight(BW)(g)	588.6±10.5	562.8±29.46	394.0±17.79*	425.8±18.75
Liver weight index(%)	2.39±0.41	2.8±0.14	7.13±0.47*	5.87±0.80▴
EAW/BW(%)	1.97±0.27	2.07±0.26	1.02±0.23*	1.6±0.36▴

*P*<0.01 *vs.* the control group and the control-cele group;

*P*<0.05 *vs.* the T_2_DM-NASH group.

### Effect of celecoxib on serum metabolic parameters

Rats in the T2DM-NASH group exhibited impaired metabolic function as shown by increased serum glucose, triglyceride, LDL-cholesterol, and decreased HDL-cholesterol concentrations. In the T2DM-NASH-Cele group, celecoxib improved and reduced these observed values and had lower concentrations compared with the T2DM-NASH group, except for serum HDL-cholesterol (Table 2). Our work noted serum ALT and AST concentrations were extremely elevated in rats fed with a HF-HS diet, which further confirmed the model of steatohepatitis. In the T2DM-NASH-

**Table 2:** Serum metabolic parameters in four groups.

	CHOW diet		HF-HS diet	
Group	control	control-cele	T_2_DM-NASH	T_2_DM-NASH-Cele
Serum glucose(mmol/L)	6.25±1.28	6.15±0.70	31.21±0.86*	22.29±0.82▵
Serum insulin(mIU/L)	15.31±1.47	14.51±1.3	28.66±3.97*	19.56±3.09▵
ITT (glucose AUC) (%change from the T2DM-NASH group)	17.65±2.52	17.03±3.01	100.00±3.58*	68.71±3.76▵
Serum triglycerides(mmol/L)	0.18±0.08	0.26±0.03	0.91±0.04*	0.49±0.08▵
Serum LDL-cholesterol (mmol/L)	0.24±0.07	0.30±0.08	11.94±0.21*	2.31±0.85▵
Serum HDL-cholesterol (mmol/L)	1.24±0.17	1.11±0.10	0.94±0.07	1.11±0.11
Serum ALT(U/L)	33.00±4.06	36.8±2.39	301.80±5.40*	88.80±13.07▵
Serum AST(U/L)	76.6±7.64	80.2±6.42	345.4±9.63*	99.8±20.51▵

*P*<0.01 *vs.* the control group and the control-cele group;

*P*<0.01 *vs.* the T_2_DM-NASH group.

Cele group, celecoxib treatment significantly lowered serum ALT and AST concentrations compared with the T2DM-NASH group (Table 2).

### Celecoxib treatment ameliorated insulin sensitivity

**Figure 1:**
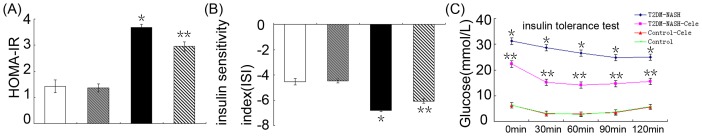
Assessment of insulin resistance in the four groups. HOMA-IR(A); Insulin sensitivity index(ISI) (B); insulin tolerance test (ITT) (C). ^*^ *P*<0.01 *vs.* the control group and control-cele group; ^**^ *P*<0.01 *vs.* the T2DM-NASH group. White = control; Gray = control-cele; Black = T2DM-NASH; Striped = T2DM-NASH-Cele.

Insulin resistance was induced in the T2DM-NASH group as shown by hyperinsulinemia, increased HOMA-IR, and decreased insulin sensitivity index (ISI) as compared with the control group and the control-cele group (Table 2; Fig. 1A and B). Compared to the T2DM-NASH group, celecoxib significantly reduced insulin levels and HOMA-IR and increased ISI in the T2DM-NASH-Cele group (Table 2; Fig. 1A and B). Furthermore, the T2DM-NASH group rats displayed reduced response to exogenous insulin in the insulin tolerance test (ITT) and increased glucose AUC value by about 82% than the control and control-cele groups (Table 2; Fig. 1C). Treatment with celecoxib significantly improved the insulin sensitivity and reduced the glucose AUC by about 31% in the T2DM-NASH-Cele group (Table 2; Fig. 1C).

### T2DM rats fed with a HF-HS diet manifested NASH and celecoxib reversed NASH

**Figure 2:**
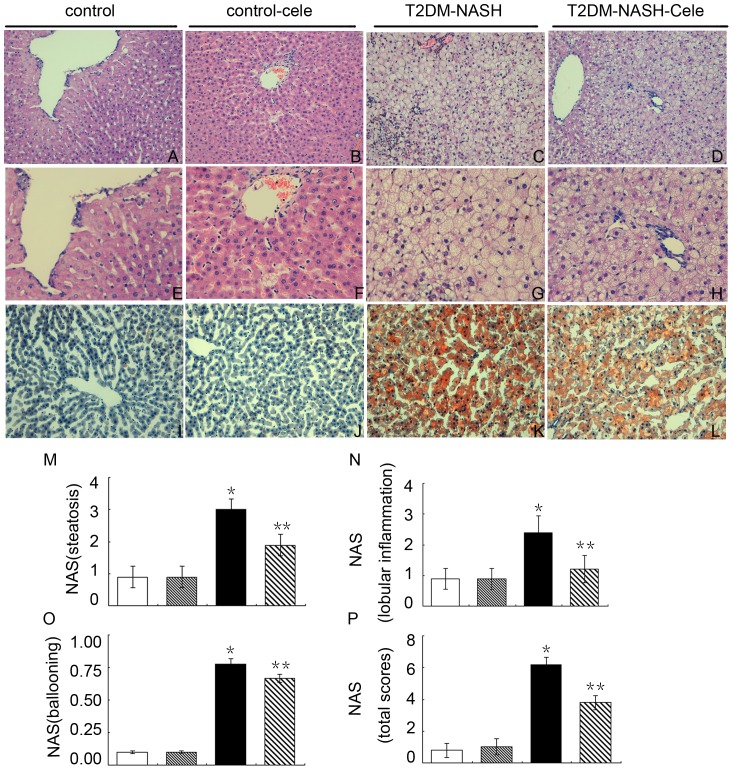
T_2_DM rats fed with a HF-HS diet developed severe steatohepatitis. H&E-stained livers ((A–D; ×200) and (E–H; ×400)). Oil Red-O staining of livers lipid accumulation (I–L; ×200). The results of NAFLD activity score (NAS) (M–P). Quantification results of steatosis (M), lobular inflammation (N), ballooning (O), NAS total scores (P). ^*^ *P*<0.01 *vs.* the control group and the control-cele group;^**^ *P*<0.01 *vs.* the T2DM-NASH group. White = control; Gray = control-cele; Black = T2DM-NASH; Striped = T2DM-NASH-Cele.

The control group had normal liver morphology and no steatosis (Fig. 2A, E and I), the same as the control-cele group (Fig. 2B, F and J). The T2DM-NASH group exhibited significant non-alcoholic steatohepatitis by histological examination (Fig. 2C, G and K). However, the T2DM-NASH-Cele group appeared mild steatotic and steatohepatitis by histological examination that was comparable with the T2DM-NASH group (Fig. 2D, H and L). By the method of the NAFLD activity score (NAS) liver sections from the T2DM-NASH group showed severe steatosis, moderate lobular inflammation, and diffuse hepatocyte ballooning; NAS (total scores) was greater than four. In the celecoxib treatment group, steatosis, lobular inflammation, and NAS (total scores) were markedly reduced; however, celecoxib didn't make a significant change of ballooning degeneration compared with the T2DM-NASH group (Fig. 2M, N, O and P).

### Increased Wnt5a/JNK1 expression in liver tissues from the T2DM-NASH group and suppressed by celecoxib

**Figure 3:**
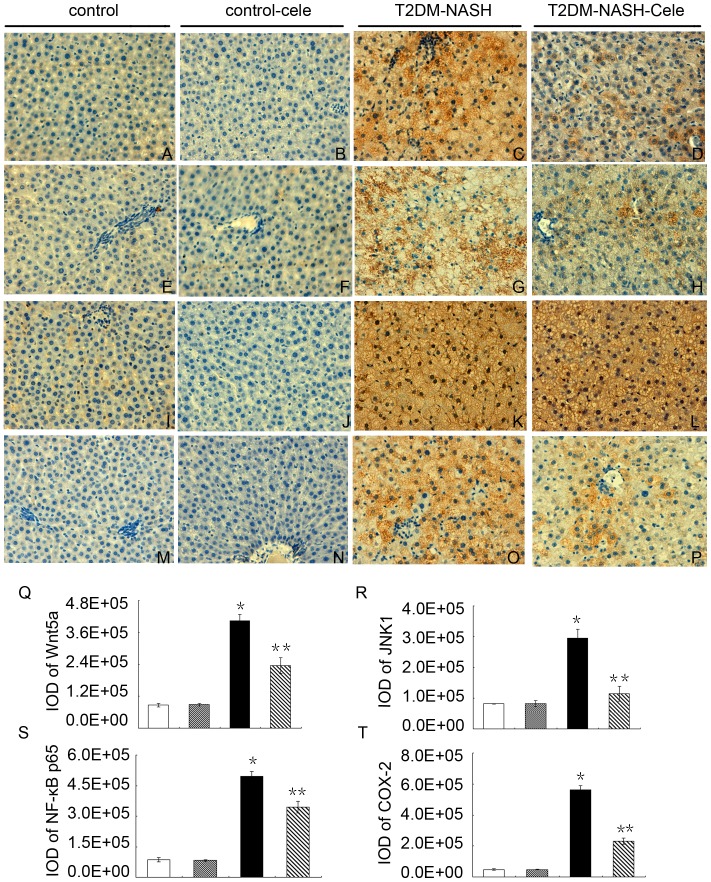
Immunohistochemistry(IHC) staining for Wnt5a, JNK1, NF-κB p65, and COX-2. IHC staining for Wnt5a (A–D; ×400); IHC staining for JNK1 (E–H; ×400); IHC staining for NF-κB p65 (I–L; ×400); IHC staining for COX-2 (M–P; ×400). The Integral Optical Density(IOD) of Wnt5a (Q), JNK1 (R), NF-κB p65 (S); COX-2 (T). ^*^ *P*<0.01 *vs.* the control group and the control-cele group; ^**^ *P*<0.01 *vs.* the T2DM-NASH group. White = control; Gray = control-cele; Black = T2DM-NASH; Striped = T2DM-NASH-Cele.

**Figure 4:**
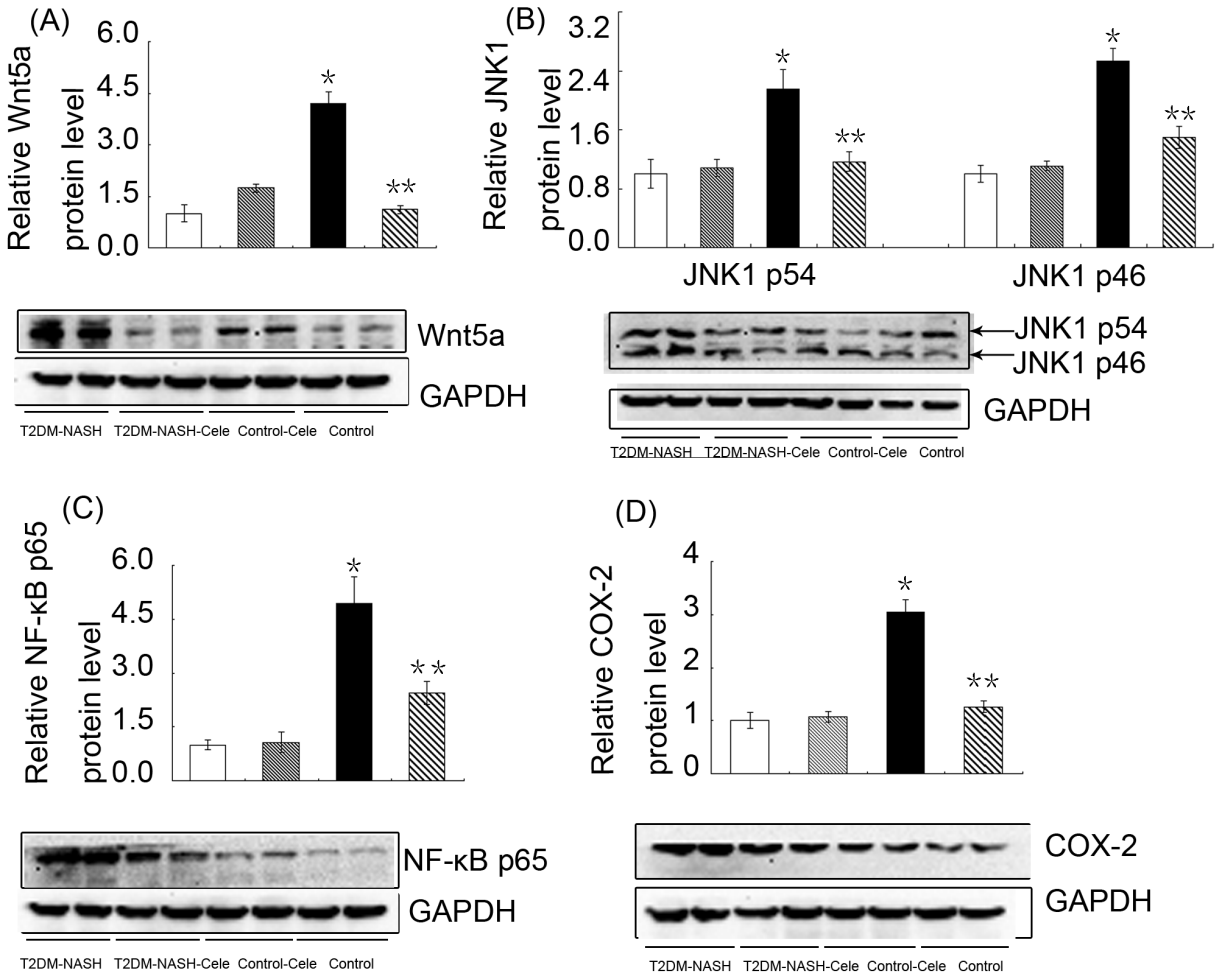
Western blot analysis of Wnt5a, JNK1, NF-κB p65, and COX-2 protein expression. Representative western blot images and quantitative analysis of Wnt5a (A), JNK1 p54 and p46 (B), NF-κB p65 (C), and COX-2 (D). GAPDH was used as a loading control. ^*^ *P*<0.01 *vs.* the control group and the control-cele group; ^**^ *P*<0.01 *vs.* the T2DM-NASH group. White = control; Gray = control-cele; Black = T2DM-NASH; Striped = T2DM-NASH-Cele.

**Figure 5:**
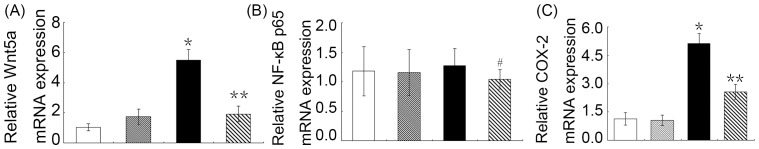
Real-time pcr analysis of Wnt5a, NF-κB p65, and COX-2 mRNA expression. Quantitative analysis of Wnt5a mRNA (A), NF-κB p65 mRNA (B), COX-2 mRNA (C). β-actin was used as a control. ^*^ *P*<0.01 *vs.* the control group and the control-cele group; ^**^ *P*<0.01 *vs.* the T2DM-NASH group. ^#^ *P*<0.05 *vs.* the T2DM-NASH group. White = control; Gray = control-cele; Black = T2DM-NASH; Striped = T2DM-NASH-Cele.

To test whether hepatic Wnt5a/JNK1 pathway involves in the mechanism of NASH, we examined their expression by different methods. IHC staining for Wnt5a and JNK1 in livers from the T2DM-NASH group were both strongly positive (Fig. 3C and G), whereas they were almost negative in the control group (Fig. 3A and E) and the control-cele group (Fig. 3B and F). The Integral Optical Density (IOD) of Wnt5a and JNK1 in the T2DM-NASH group were both significantly higher than those in the control goup and the control-cele group (Figure 3Q and R). Celecoxib treatment significantly reduced the IOD of Wnt5a and JNK1 in the T2DM-NASH-Cele group (Figure 3Q and R). Western blot analysis showed that hepatic Wnt5a, JNK1 p54 and JNK1 p46 protein levels in the T2DM-NASH group were significantly increased compared with those of the control group and control-cele group (Fig. 4A and B), as was hepatic Wnt5a mRNA (Fig. 5A). Consistent with the protective effect, celecoxib administration supressed the expression of Wnt5a and JNK1. Hepatic Wnt5a, JNK1 p54 and JNK1 p46 protein levels of the T2DM-NASH-Cele group were all strongly reduced than those from the T2DM-NASH group (Fig. 4A and B), as was hepatic Wnt5a mRNA (Fig. 5A).

### NF-κB and COX-2 were activated in liver tissues from the T2DM-NASH group and were reduced by celecoxib

We tested whether NF-κB was activated in the model of T2DM-NASH. Consistent with the current findings, hepatic NF-κB p65 protein level was significantly increased in the T2DM-NASH group (Fig. 3K and S; Fig. 4C) compared with the control group (Fig. 3I and M) and the control-cele group(Fig. 3J and N). Celecoxib determined a decrease of the NF-κB p65 protein in the T2DM-NASH-Cele group (Fig. 3L and S; Fig. 4C). In the T2DM-NASH group, more hepatocytes nucleus were stained (Fig. 3K). In the T2DM-NASH-Cele group, the nuclei were slightly stained (Fig. 3L), which indicated that celecoxib administration supressed nuclear translocation of NF-κB p65. However, we detected no significant difference of NF-κB p65 mRNA in the T2DM-NASH group (Fig. 5B) compared with the control group and the control-cele group. Celecoxib just slightly reduced NF-κB p65 mRNA in the T2DM-NASH-Cele group (Fig. 5B). The IHC and Western blotting showed that hepatic COX-2 protein level was significantly increased in the T2DM-NASH group (Fig. 3O and T; Fig. 4D), as was hepatic COX-2 mRNA (Fig. 5C). Hepatic COX-2 protein and mRNA levels were both obviously decreased by celecoxib in the T2DM-NASH-Cele group (Fig. 3P and T; Fig. 4D; Fig. 5C).

## Discussion

In this study, a HF-HS diet added to T2DM induction was shown to cause the model of diabetes-related NASH where both the pathophysiology and liver pathology closely resembled human NASH established with T2DM. In the T2DM-NASH group, while hyperinsulinemia was observed to indicate the predomination of insulin resistance, hyperglycemia showed relative insulin deficiency. Insulin resistance and insulin deficiency of adipose tissue cause increased central lipolysis and elevated circulating free fatty acids, with resulting increased hepatic delivery and uptake [17]. In the T2DM-NASH group we observed a marked reduction of EAW/BW and a significant increase of liver weight index, implicating enhanced adipose lipolysis and hepatic intake. The increased circulating triglyceride and LDL-cholesterol were consistent with increased lipolysis. As expected, we observed decreased HDL-cholesterol in the T2DM-NASH group, which is the characteristic of NAFLD [18].

An early study has shown that high doses of salicylates, a kind of Nonsteroidal anti-inflammatory drugs (NSAIDs) reverse hyperglycemia, hyperinsulinemia, and dyslipidemia in obese rodents by sensitizing insulin signaling [19]. Hence, we detected the role of celecoxib, an NSAID that is a selective COX-2 inhibitor, on the development of T2DM-related NASH. In the T2DM-NASH-Cele group, we observed that celecoxib weakened central lipolysis indicated by increased EAW/BW and decreased liver weight index, compared with the T2DM-NASH group.Besides, we observed that celecoxib ameliorated insulin resistence, including reduced serum insulin level, HOMA-IR, glucose AUC and increased insulin sensitivity in the T2DM-NASH-Cele group. Also, celecoxib ameliorated dyslipidemia, including reduction of serum triglyceride and LDL-cholesterol levels. However, there was no obvious effect on HDL-cholesterol. The mechanism about the improvement of dyslipidemia needs to be further explored.Another benefit of celecoxib in our data were considerable improvement of liver steatosis and inflammation indicated by reduction of NAFLD activity score (NAS). Celecoxib alle-viated liver injury showed by reduction of serum ALT and AST levels. All of these events might be causally related to changes of cytokines we tested.

We monitored COX-2 expression because it is a key enzyme in activating the inflammatory response. And inflammation caused by COX-2 activation in fat and liver tissue plays a pivotal role in the development of insulin resistance and NAFLD [6], [8]. The present study further examined that the driving effect of COX-2 mediated inflammation in the development of NASH in diabetic rats. Alternatively, celecoxib reduced COX-2 expression and ameliorated NASH, consistently with the current literatures. Earlier studies identified NF-κB as a key regulator of hepatic inflammatory recruitment and liver injury in NASH [20], [21]. COX-2 is regulated by NF-κB because COX-2 gene promoter has two binding sites for NF-κB which works as a positive regulator of COX-2 gene during inflammatory response [22]. Also, COX-2 inhibitors had been observed to suppress COX-2 expression through NF-κB inactivation pathway [23]. And in our research, we found that NF-κB protein level was significantly increased in the T2DM-NASH group, and nuclear translocation of p65 was reduced by celecoxib in the T2DM-NASH-Cele group. However, NF-κB mRNA level had no significant difference. Possibly, it didn't respond adequately to metabolic stress at the gene level.

Recently, the role of Wnt signaling pathway facing to the metabolic stress has been paid for attention. The canonical Wnt/β-catenin signaling resists adipogenesis and improves glucose tolerance and insulin sensitivity [24]–[27]. Schulte DM et al [28] showed that the non-canonical Wnt5a might act as an important pro-inflammatory molecule in low grade inflammation of adipose tissue in obese humans. However, the role of Wnt signaling pathway in NAFLD is less researched. The canonical Wnt/β-catenin signaling may confer a protective effect on the liver in the face of metabolic stress. Hepatocyte-specific loss of β-catenin led to increased susceptibility to developing steatohepatitis and fibrosis in the mice fed with the MCD diet [12]. The protection mechanism could be the role of β-catenin maintaining mitochondrial function and regulating ATP production, which is the principle energy source for lipid and glucose oxidative metabolism [29], [30]. However, little is known about the role of non-canonical Wnt5a signaling pathway on the development of NASH. These observations prompt us to investigate changes of Wnt5a expression in the liver. To our knowledge, this is the first report that shows a significant correlation between hepatic increased Wnt5a protein and mRNA expression and development of NASH, especially associated with T2DM in the liver tissue. JNK was a downstream target of the non-canonical Wnt signaling pathway [31]. Activation of JNK1 is associated with hepatic triglyceride accumulation and insulin resistance in rats fed a high-fat diet [32]. Ouchi et al [13] showed that Wnt5a upregulated JNK1 expression in fat tissues and exacerbated hepatic steatosis in obesity mice. Inhibition of Wnt5a/JNK1 axis improved insulin sensitivity and metabolic function. Consistently, in our study, we discovered that the protein levels of JNK1 at p54 and p46 subunit were both significantly increased in the T2DM-NASH group. All the data indicated the non-canonical Wnt5a/JNK1 signaling was highly expressed in the model of T2DM-related NASH, probably involving in the process of development of T2DM-related NASH.

Another striking finding was that celecoxib significantly reduced the expression of Wnt5a at both protein and mRNA level accompanied with protective effect of celecoxib on the development of T2DM-related NASH in the T2DM-NASH-Cele group. As for JNK1, celecoxib decreased the levels of JNK1 at p54 and p46 subunit in the T2DM-NASH-Cele group, consistently with the current study [33]. Our current data indicated that protective effect of celecoxib was probably related to its suppression of the Wnt5a and JNK1 expression and there was interaction between celecoxib and the Wnt5a/JNK1 signaling. Recent reports showed that there was direct synergetic interaction between COX-2 and Wnt/β-catenin signaling cascades. Likely, in the COX-2 promoter there was TBE Site II and β-catenin could bind to this promoter region to regulate COX-2 expression [34]. And one of the bioactive products of COX-2, prostaglandin E2 could activate components of the canonical Wnt signaling through inducing phosphorylation of GSK-3β, ultimately promoting the activity of β-catenin [35], [36]. Celecoxib can inhibit Wnt/β-catenin activity through inducing decreased activity of GSK-3β [37]. However, there is no research about the crosstalk between non-canonical Wnt signaling and COX-2. In our research, we found that increased expression of Wnt5a and COX-2 were in parallel in the liver of T2DM-NASH rats, which can be both reduced by celecoxib. These observations demonstrate that COX-2 is directly involved in the regulation of Wnt5a by celecoxib. We supposed NF-κB might play the bridge role on connecting Wnt5a and COX-2. NF-κB can upregulate Wnt5a because there is NF-κB-binding site within Wnt5a promoter B [14]. And Wnt5a can induce NF-κB activity through Wnt5a/ROR1/Ca^2+^ pathway [15]. Herein, we inferred that increased Wnt5a upregulated COX-2 expression to mediate hepatic inflammation by activating NF-κB. And celecoxib might reduce Wnt5a through inactivating NF-κB. The molecular mechanisms underlying remain to be elucidated.

In summary, we first identified increased hepatic Wnt5a expression accompanying activation of JNK1, NF-κB, and COX-2 was associated with the development of T2DM-NASH. Treatment with the selective COX-2 inhibitor celecoxib ameliorated T2DM-related NASH as shown by anti-inflammatory activity and restraining insulin resistance, probably via suppression of the non-canonical Wnt5a/JNK1 signaling pathway and NF-κB. The mechanism by which celecoxib regulates Wnt5a expression via COX-2 needs further investigation and our results may suggest a new molecule mechanism in therapy strategy.
